# The Role of Monoclonal Antibody Targeted Therapy (Panitumumab) in the Treatment of Metastatic Colorectal Cancer—A Narrative Review of Current Evidence and Emerging Therapeutic Strategies

**DOI:** 10.3390/ijms27177559

**Published:** 2026-08-24

**Authors:** Lidia Kwiatkowska, Małgorzata Szczuko

**Affiliations:** Department of Bromatology and Nutritional Diagnostics, Pomeranian Medical University in Szczecin, 70-111 Szczecin, Poland; malgorzata.szczuko@pum.edu.pl

**Keywords:** metastatic colorectal cancer, *KRAS*, MSI, *BRAF*, HER2, ctDNA, panitumumab, anti-EGFR

## Abstract

Metastatic colorectal cancer (mCRC) remains one of the leading causes of cancer deaths worldwide. Mutations of the *RAS* and *BRAF* genes and the location of the primary tumor determine the choice of systemic therapy. Panitumumab—a fully human anti-EGFR monoclonal antibody—is well-established in the treatment of patients with wild-type *RAS* and *BRAF* and left-sided tumor localization. The aim of this review is to summarize the role and potential of targeted therapies for colorectal cancer with monoclonal antibodies such as panitumumab or cetuximab, to provide current evidence on the position of panitumumab in the treatment of mCRC, and to discuss biomarkers and qualification strategies for targeted therapy, including the role of ctDNA in disease monitoring and rechallenge. The work is based on a narrative review of literature from the PubMed, Scopus, Web of Science and Google Scholar databases, including publications available until May 2026. The efficacy of panitumumab was confirmed in phase III studies: first-line (PRIME: prolongation of OS by 5.6 months) and second-line treatment. A key condition for eligibility is complete *RAS* and *BRAF* genotyping, assessment of MSI/dMMR status, HER2 amplification, and tumor location. ctDNA analysis enables monitoring of tumor clonal evolution and qualification for rechallenge—the CHRONOS study showed 30% objective responses with liquid biopsy-based selection. Associations with *BRAF*/MEK and *KRAS* G12C inhibitors and modulation of the immune microenvironment via the *CCL5*/*CCR5* axis remain promising directions. Panitumumab occupies an important position in the treatment of patients with mCRC, but increasingly precise molecular stratification and dynamic monitoring of disease with ctDNA are becoming the basis for the effective and rational use of the drug. Unlike reviews that selectively focus on individual aspects of anti-EGFR therapy, the following work integrates all aspects: from molecular biology and biomarkers used to qualify patients to clinical data on panitumumab and new strategies in the fight against mCRC. Further development of next-generation sequencing methods and standardization of ctDNA panels will be crucial for the widespread deployment of targeted therapies in everyday clinical practice. The role of panitumumab is well established in appropriately selected patients with RAS wild-type, particularly left-sided, metastatic colorectal cancer (mCRC). Combining panitumumab with immune checkpoint inhibitors warrants further investigation, particularly in patients with MSS/pMMR mCRC. The observed activity of panitumumab in combination with nivolumab and ipilimumab provides a rationale for further clinical evaluation and monitoring.

## 1. Introduction—Epidemiology

Colorectal cancer is the third most common cancer in the world, accounting for about 10% of all cancer cases. It is also the second most common cause of oncological death. Colorectal cancer includes colon and rectal cancers, most often originating from the epithelium of the intestinal mucosa, and metastatic disease is the stage at which the cancer has spread beyond its original location to distant organs. The disease mainly affects people over 50 years of age, but in recent years there has been an increase in incidence among younger populations, likely due to more effective patient diagnosis. The five-year survival rate is about 65%, while in the metastatic stage it falls below 15% [1]. The most important risk factors include a diet rich in red and processed meat, obesity, lack of physical activity, smoking, and alcohol consumption [2]. Many of these factors share a common mechanism —chronic damage and inflammation of the intestinal mucosa, which promote the accumulation of genetic changes that lead to tumor transformation. Importance is also attributed to disorders of the intestinal microbiota. A special role in colorectal cancer carcinogenesis is attributed to bacteria such as Fusobacterium nucleatum, Bacteroides fragilis and *Escherichia coli*. They promote tumour development by inducing chronic inflammation, modulating the immune response, metabolic reprogramming of host cells, and direct genotoxic effects [3]. Despite the rapid development of immunotherapy and novel targeted agents, anti-EGFR monoclonal antibodies remain one of the cornerstones of precision treatment for selected patients with metastatic colorectal cancer.

Early detection of the disease remains crucial, as a significant proportion of patients do not report symptoms in the early stages [4]. Metastases occur in about 20–25% of patients at the time of diagnosis and are the main cause of death. mCRC causes metastases mainly to the liver, lungs, and peritoneal cavity, and less often to the brain, bones, or adrenal glands [5]. Understanding these metastasis pathways is clinically important as the location of metastases influences the choice of treatment strategy. Advances in understanding the molecular biology of mCRC over the past two decades have led to a shift from a uniform therapeutic approach to a targeted model in which treatment selection is based on the individual tumor genetic profile. Mutations in the *KRAS*, *NRAS* and *BRAF* genes have a significant impact on the course of the disease and the effectiveness of treatment (Kirsten RAS proto-oncogenes and neuroblastoma RAS, protein kinase B-Raf) [6,7]. Therefore, molecular profiling has become an essential component of treatment planning in mCRC. In addition to *RAS* and *BRAF* mutational status, contemporary molecular assessment incorporates MSI/dMMR status and, in selected clinical settings, HER2 and other actionable alterations. Current recommendations for molecular biomarker testing according to NCCN, ESMO, and CSCO guidelines are summarized in Table 1.

## 2. Materials and Methods

This study was conducted as a narrative literature review aimed at summarizing current evidence regarding the role of panitumumab in the treatment of metastatic colorectal cancer (mCRC), with particular emphasis on patient selection, predictive biomarkers, mechanisms of resistance, and emerging therapeutic strategies.

A comprehensive literature search was performed using the PubMed, Web of Science, Scopus, and Google Scholar databases. Publications available up to May 2026 were considered. The search strategy combined Medical Subject Headings (MeSHs) and free-text keywords, including metastatic colorectal cancer, panitumumab, cetuximab, anti-EGFR therapy, *RAS* mutation, *KRAS*, *NRAS*, *BRAF*, HER2, MSI, mismatch repair, circulating tumor DNA, ctDNA, liquid biopsy, rechallenge, precision oncology, EGFR resistance, *CCR5*, maraviroc, tumor microenvironment, and immunotherapy. Search terms were combined using the Boolean operators AND and OR and adapted to the syntax of each database. For example, the core PubMed search strategy combined (“colorectal cancer” OR “metastatic colorectal cancer”) AND (“panitumumab” OR “anti-EGFR”) with topic-specific terms including (“*RAS*” OR “*KRAS*” OR “*NRAS*” OR “*BRAF*” OR “HER2” OR “MSI” OR “ctDNA” OR “liquid biopsy” OR “rechallenge” OR “resistance”). Reference lists of relevant reviews, guidelines, and pivotal clinical trials were additionally screened to identify potentially eligible publications not captured by the database search.

Priority was given to phase II and III randomized clinical trials, systematic reviews, meta-analyses, international clinical practice guidelines (NCCN, ESMO, CSCO, and FDA) and regulatory documents and high-quality original studies published in peer-reviewed journals. In addition, selected preclinical studies investigating novel mechanisms of resistance and emerging therapeutic targets were included when considered relevant to understanding current and future treatment strategies.

Studies directly evaluating panitumumab were prioritized over studies evaluating anti-EGFR therapy more broadly. Evidence derived from cetuximab-based studies was included only when necessary to discuss class-level mechanisms, molecularly defined treatment strategies, or areas in which panitumumab-specific evidence remains limited; such evidence was not considered direct evidence of panitumumab efficacy.

Publications not available as full-text articles, conference abstracts without subsequent peer-reviewed publication, case reports, and studies published in languages other than English were excluded. One recently published preprint [11] addressing the potential role of leronlimab in metastatic colorectal cancer was included because of its relevance to emerging therapeutic strategies; however, its preliminary nature and lack of peer review are clearly acknowledged in the Discussion. Because this was a narrative rather than a systematic review, no formal risk-of-bias or GRADE assessment was performed. Instead, evidence was critically appraised according to study design, phase, sample size, molecular selection criteria, comparator, clinical endpoints, and direct relevance to panitumumab. Randomized phase III trials were considered the principal source of clinical efficacy evidence, followed by prospective phase II studies and retrospective analyses. Systematic reviews, meta-analyses, and current clinical practice guidelines were used to contextualize individual study findings, whereas preclinical studies and non-peer-reviewed evidence were considered hypothesis-generating. Particular attention was paid to distinguishing direct panitumumab-specific evidence from findings concerning the anti-EGFR drug class as a whole.

The retrieved literature was critically evaluated and organized into thematic sections. Given the narrative design, study selection was based on predefined relevance and quality criteria rather than a PRISMA-based systematic screening process. The final bibliography includes 72 items.

**Figure 1:**
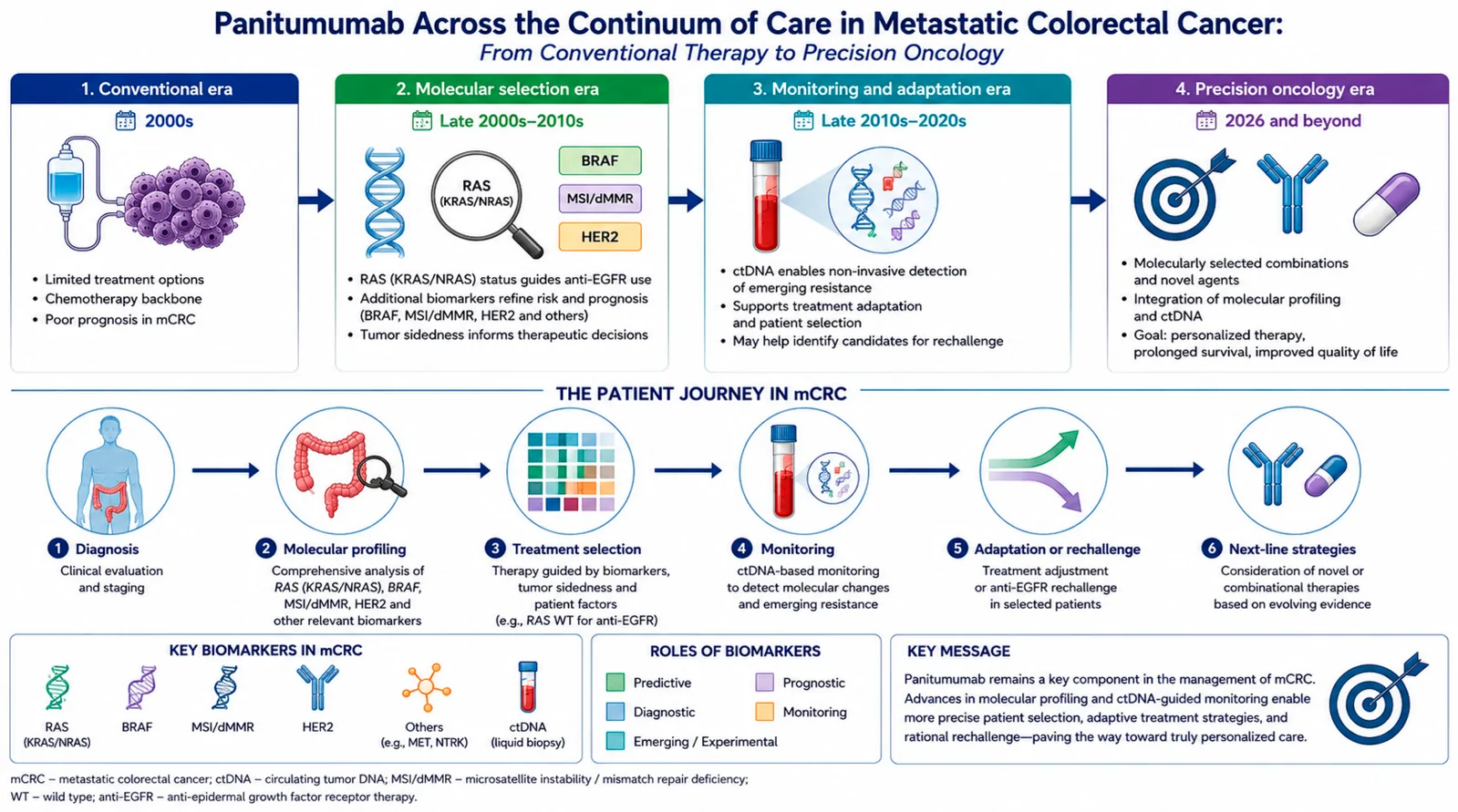
Evolution of Precision Oncology in Metastatic Colorectal Cancer: The Changing Role of Panitumumab from Conventional Anti-EGFR Therapy to Biomarker-Guided Precision Medicine.

**Figure 2:**
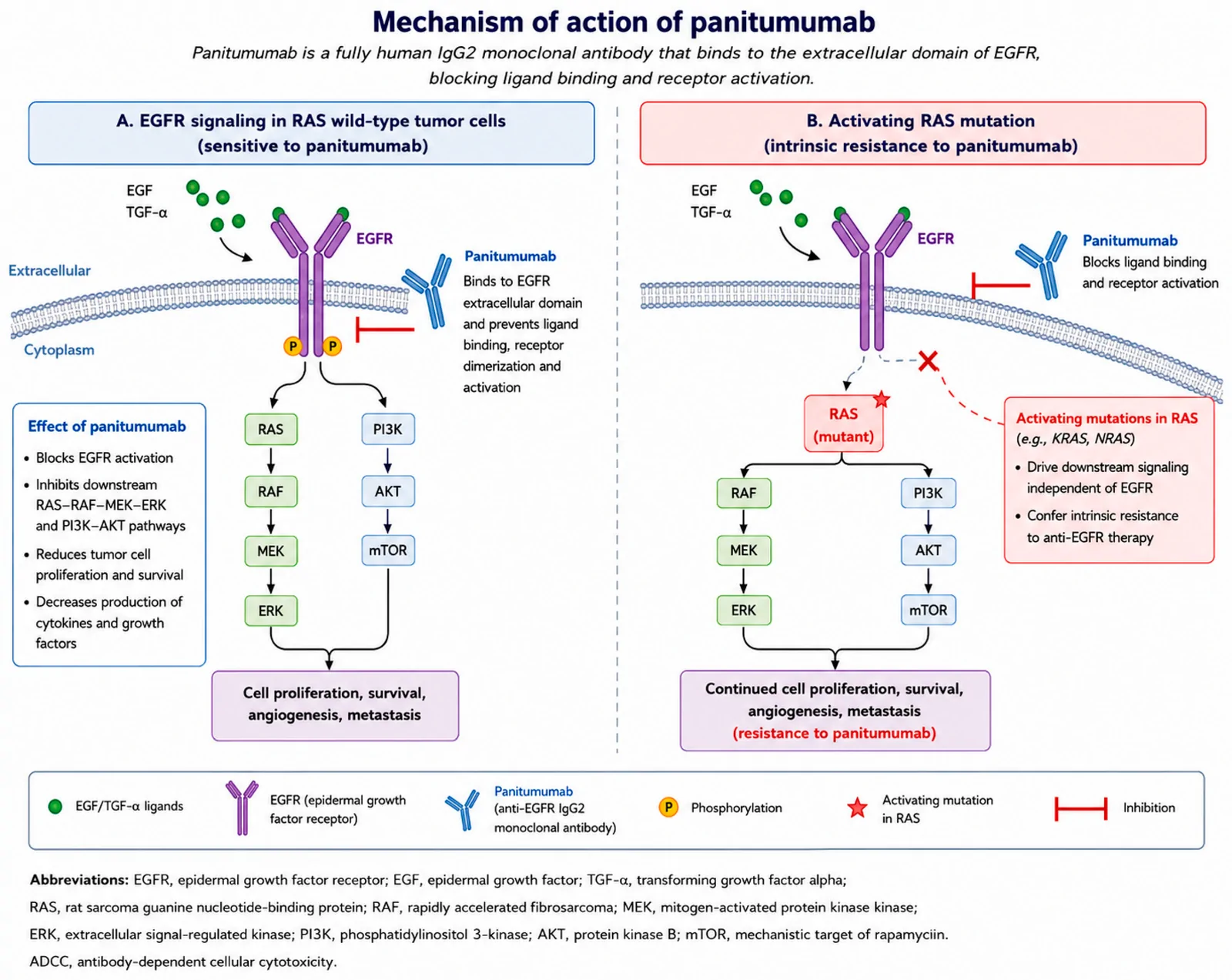
Mechanism of action of panitumumab and the molecular basis of resistance associated with activating RAS mutations.

**Figure 3:**
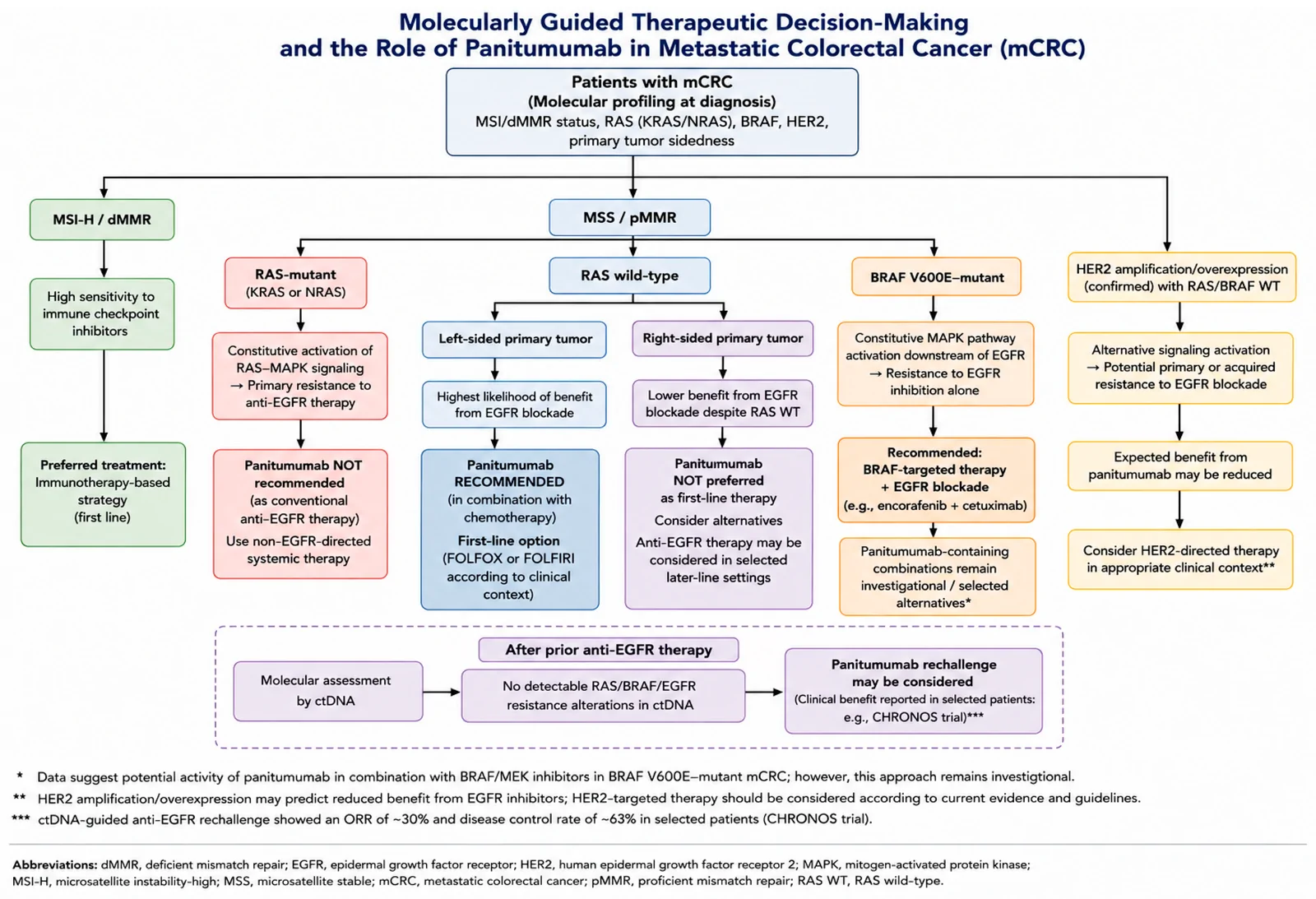
Molecularly guided therapeutic decision-making and the role of panitumumab in metastatic colorectal cancer.

Generative artificial intelligence was used to assist in the preparation of Figure 1, Figure 2 and Figure 3. The figures were subsequently reviewed and verified by the authors for scientific accuracy and clarity.

## 3. Biology of Colorectal Cancer

Colorectal cancer develops as a result of a gradual, pathological transformation of the colon epithelium—first into adenomatous polyps and then into invasive cancer. This process is driven by the accumulation of numerous genetic and molecular changes that disrupt the control of cancer cell proliferation, differentiation and survival. In recent years, therapeutic advances have increased the median survival of patients with metastatic colorectal cancer (mCRC) from 12 to 21 months, thanks in part to therapies targeting epidermal growth factor receptor (EGFR), specifically cetuximab and panitumumab [6]. These are monoclonal antibodies directed against the aforementioned epidermal growth factor receptor, with cetuximab being a chimeric monoclonal antibody and panitumumab being a fully human antibody.

A central role in the pathogenesis of CRC is played by dysregulation of the *KRAS* proto-oncogene, which encodes a signaling protein belonging to the small GTPase family. The name *KRAS* derives from the Kirsten rat sarcoma virus, in which the viral *v-Kras* oncogene was originally identified; however, *KRAS* itself is a human proto-oncogene. The KRAS protein regulates key cellular processes: proliferation, differentiation, and survival, especially in epithelial cells, vascular endothelium, and fibroblasts. Mutations in *KRAS* are found in approximately 30–50% of patients with CRC, most commonly affecting exon 2 (codons 12 and 13) and leading to constant, ligand-independent activation of the signaling pathway by significantly reducing GTPase activity. Mutations in codon 12 or 13 of exon 2 of the *KRAS* gene have been shown to predict non-response to EGFR inhibitor treatment. On the other hand, patients with wild-type *RAS* (WT) have clear clinical benefits from anti-EGFR therapy. Later studies confirmed that mutations in *KRAS* exon 2 and in the *NRAS* gene can confer resistance to cetuximab and panitumumab. For this reason, current guidelines from cancer societies recommend complete genotyping of tumor tissue—covering all relevant *RAS* gene exons and the *BRAF* gene before deciding to include anti-EGFR therapy. However, it is worth noting that some cancers with a wild-type *KRAS* still do not respond to treatment, indicating the need for further research into additional predictive biomarkers [6].

Mutation is also of significant prognostic importance in *BRAF* V600E (Val600Glu), resulting in constitutive activation of BRAF kinase and continuous activation of the MAPK pathway independently of EGFR signals. Patients with this mutation tend to have a poorer prognosis, shorter overall survival time, and limited sensitivity to targeted therapies. The exact role of *BRAF* mutations in the context of therapeutic resistance remains the subject of active research. It has also been noted that mutations in exons 3 and 4 of the *KRAS* gene are associated with a worse prognosis compared to WT cancers, which highlights the need for a broader understanding of the frequency and prognostic significance of individual mutation variants [6].

In addition to mutations in genes from the *RAS* and *BRAF* families, disorders of suppressor genes are also involved in the pathogenesis of colorectal cancer [2], such as *APC* (a key tumor suppressor gene) and *TP53*, as well as loss of the chromosomal region 18q. These changes lead to impaired cell cycle control, genetic instability, and cancer progression [2].

Biological differences between cancers of the right and left half of the colon have also become increasingly important, as differences in clinical outcomes and responses to drugs have emerged. Tumors of the proximal part of the large intestine are more likely to exhibit microsatellite instability (MSI), CpG island hypermethylation phenotype (CIMP), and *KRAS* and *BRAF* mutations. They are also usually associated with a worse prognosis. On the other hand, cancers of the distal part of the large intestine are more often characterized by chromosomal instability (CIN) and a more favorable clinical course. These differences may result from the different embryonic origins of individual sections of the intestine, as well as the influence of the microbiome, environmental factors, and local immune mechanisms [12]. The right part of the colon originates in the midgut and is vascularized by the superior mesenteric artery, while the left part of the colon and rectum originate in the posterior segment of the primary intestine and are supplied by the inferior mesenteric artery—which translates into a different physiology of the two segments: the right side is mainly responsible for the absorption of water and anaerobic digestion of the intestinal contents, while the left side is responsible for storing and moving fecal masses. This promotes distinct bacterial microbiota profiles on both sides of the colon. There are also some immunological differences. Lymphocyte populations in tumor tissue differ in abundance between right- and left-sided tumors, which corresponds to a higher incidence of MSI and *BRAF* mutations on the right side [12]. The location of the primary tumor is therefore not only an anatomical feature, but reflects the different molecular, microbiotic and immune profile of the tumor, which translates into differences in prognosis and response to treatment.

## 4. Biomarkers

Biomarkers in colorectal cancer are an important diagnostic and prognostic tool. For the purposes of a comprehensive approach to the problem, the most important of them in the context of available therapies will be discussed. Mutations in *RAS* and *BRAF* were discussed earlier. Molecular genotyping of *KRAS*, *NRAS*, and *BRAF* can be performed using various validated molecular methods, including PCR-based assays and next-generation sequencing (NGS). According to the recommendations of the NCCN (National Comprehensive Cancer Network), the examination should be performed from primary or metastatic material. Activating *RAS* mutations is clinically relevant because they predict resistance to conventional anti-EGFR therapy [7]. Colorectal cancer is also characterized by the occurrence of MSI (microsatellite instability) at varying rates depending on the cancer. MSI is based on the accumulation of errors in short, repetitive DNA sequences resulting from a defect in the mismatch repair (MMR) mechanism. There are MSI-H, MSI-L and MSS. MSI-H tumors are characterized by a higher number of mutations, which is of significant diagnostic importance [13]. MSI-L behaves similarly to MSS, i.e., those with microsatellite stability. MSS affects most mCRCs and involves standard management based on chemotherapy and targeted therapies (anti-EGFR, anti-VEGF). However, MSS status is not an absolute marker of nonresponse to immunotherapy, as some of these tumors have been described with an immune profile similar to MSI-H, a subgroup of patients for which active research is underway [14,15]. dMMR/MSI-H occurs in approximately 15% of non-metastatic CRCs but is less frequent in metastatic disease, where it is observed in approximately 4–5% of cases [7].

Liquid biopsy based on the analysis of circulating free tumor DNA (ctDNA) is a non-invasive diagnostic and monitoring tool in metastatic colorectal cancer. ctDNA reflects the tumor’s current molecular profile, enabling real-time assessment of its heterogeneity [16]. It is used to identify key genetic changes, including *RAS* and *BRAF* mutations, and to detect the mechanisms of acquired resistance to treatment [17]. Its level has been shown to correlate with the prognosis. An increase in ctDNA during therapy is associated with disease progression and shortened survival [18]; thus, ctDNA is considered one of the key biomarkers of modern precision medicine in mCRC [16,19].

Tumor Mutational Burden (TMB) determines the number of somatic mutations per megabase of sequenced DNA. High TMB increases the number of tumor neoantigens, enhancing the immune response and the effectiveness of immunotherapy. In colorectal cancer, it is most common with MSI-H/dMMR, although it also applies to some MSS cancers with *POLE* and *POLD1* mutations. TMB-high tumors are more likely to respond to checkpoint inhibitors; however, the clinical use of this biomarker remains under investigation. The main obstacle is the lack of standardization of marking methods and cut-off values. TMB is now primarily considered a predictor of response to immunotherapy, especially when combined with MSI/MMR assessment [20].

Another biomarker is the expression of the PD-L1 protein, a ligand for the programmed cell death receptor. PD-L1 expression inhibits the immune response, enabling cancer cells to survive by binding to the PD-1 receptor on T cells. Higher PD-L1 expression correlates with a worse prognosis [21]. In colorectal cancer, overexpression of HER2 is also being investigated as a potential therapeutic target. HER2 is a protein encoded by the erythroblastic oncogene B (*ERBB2*) gene and found in cell membranes. HER2 overexpression affects 3–5% of mCRCs, mainly left-sided adenocarcinomas, and is associated with a characteristic pattern of CNS (central nervous system) metastasis. HER2 overexpression often co-occurs with resistance to anti-EGFR treatment, which is why the NCCN recommends testing HER2 amplification alongside classical biomarkers [22]. Anti-HER2 therapy is indicated for wild-type *RAS* and *BRAF* [7]. The MOUNTAINEER study demonstrated the efficacy of the trastuzumab + tucatinib regimen: response rate > 39%, median duration of response 15.2 months (Phase II analysis, 2026) [23]. This regimen was the first to obtain FDA registration for this indication [24].

Proto-oncogene *MET* encodes receptor tyrosine kinase c-MET—a receptor for hepatocyte growth factor (HGF). Its amplification or overexpression leads to uncontrolled activation of the MAPK and PI3K/AKT pathways, promoting angiogenesis, tumor progression and metastasis. In mCRC, *MET* amplification is considered to be one of the factors of tumor progression, as well as resistance to anti-EGFR treatment (cetuximab, panitumumab). The study by Tomokazu Kishiki et al. showed that overexpression of MET is associated with lower disease control rates and shorter progression-free survival in patients with wild-type *KRAS* cancer [25]. An important observation is that *MET* amplification most often develops as a mechanism of acquired resistance to anti-EGFR therapy, rather than as a primary feature of the tumor—its incidence is relatively low in primary mCRC tissues, while it is markedly increasing in refractory patients. Moreover, *MET* amplification can be detected in circulating DNA even before clinical progression, highlighting its value as a predictive biomarker for monitoring the development of resistance [26]. It has also been shown that c-MET expression is higher in metastatic foci than in the primary tumor, indicating a significant role of the HGF/MET pathway in the metastasis process. Activation of this axis promotes cancer cell migration, tissue invasion, and protection against apoptosis [27]. Activation of HGF/c-MET may underlie both primary and acquired resistance to molecular therapies, making the MET pathway and its inhibitors promising therapeutic targets [28].

Fusion of *NTRK1*/*NTRK2*/*NTRK3* genes leads to the formation of constitutively active TRK fusion proteins activating proliferative pathways of cancer cells. In colorectal cancer, they occur rarely (<1% of cases), but their clinical importance is high due to the high effectiveness of TRK inhibitors. *NTRK* fusions are more commonly observed in MSI-H/dMMR tumors and are a predictive biomarker of response to molecularly targeted treatment [29].

*CCR5* is a G protein-coupled receptor primarily expressed on immune cells [30]. Activation of the *CCL5*/*CCR5* axis in mCRC enhances tumor progression by stimulating cell migration, angiogenesis, and extracellular matrix remodeling, and creates an immunosuppressive microenvironment by recruiting Tregs, macrophages, and MDSC cells—limiting the antitumor response and promoting resistance to immunotherapy [30,31]. Battaglin et al. showed a correlation between *CCR5*/*CCL5* expression and dMMR/MSI-H phenotype and PD-L1 expression, and elevated expression of this axis was more common in right-sided tumors, metastatic lesions, and rectum—confirming its role in metastatic biology and immune response, justifying CCR5 as a potential therapeutic target [31]. Maraviroc, a CCR5 antagonist, remains the subject of research in mCRC. Pervaiz et al. have shown that it inhibits proliferation, stops the cycle in the G1 phase, and induces apoptosis (in vitro study on cell lines) [32]. A phase I PICASSO study evaluating the combination of maraviroc and pembrolizumab showed a favorable toxicity profile, suggesting prospects for this strategy [33]. The principal molecular biomarkers relevant to treatment selection, prognosis, resistance monitoring, and emerging therapeutic strategies in mCRC are summarized in Table 2.

Among the biomarkers currently available, RAS mutation status remains the strongest predictor of anti-EGFR therapy response, while ctDNA emerges as the most promising dynamic biomarker for treatment monitoring. However, cost, lack of standardization, and different sensitivity of ctDNA panels are currently the biggest challenges and limitations. Only RAS/BRAF, MSI/dMMR status, and HER2 amplification (MOUNTAINEER study) directly impact registered therapies and clinical guidelines. Other biomarkers remain promising directions for development at the research stage. Figure 1 below shows the bridge between cancer biology and clinical practice.

## 5. Monoclonal Antibodies in the Treatment of mCRC—Mechanisms of Action

EGFR (HER-1), or epidermal growth factor receptor, is a transmembrane glycoprotein belonging to the family of receptor tyrosine kinases. It includes four receptors: EGFR, HER2, HER3, and HER4. EGFR is often incorrectly activated in many malignancies, including colorectal cancer, by, among others, mutations, receptor overexpression or loss of regulatory mechanisms. Activation of EGFR by its ligands triggers a cascade of signaling pathways, RAS-RAF-MAPK, PI3K-PTEN-AKT, and JAK/STAT, which regulate proliferation, cell migration, survival, angiogenesis, and metastasis. Dysregulation of these pathways is one of the key mechanisms of mCRC progression, justifying EGFR as one of the therapeutic targets [37,38]. Panitumumab is a human IgG2-class monoclonal antibody that targets the extracellular domain of EGFR. It binds specifically to EGFR on both normal and cancer cells, competitively inhibiting receptor–ligand binding. This blockade prevents receptor autophosphorylation and activation of its associated kinases, leading to inhibition of cell growth, induction of apoptosis, reduction in production of pro-inflammatory cytokines and proangiogenic factors, and internalization of EGFR [39]. The mechanism of EGFR inhibition by panitumumab and the effect of activating RAS mutations on downstream signaling are summarized in Figure 2.

Cetuximab is the second EGFR inhibitor used, a chimeric murine–human IgG1 monoclonal antibody. It activates antibody-dependent cellular cytotoxicity (ADCC) through interaction between the Fc region of the antibody and the CD16 receptor on natural killer (NK) cells. This leads to lysis of antibody-coated cancer cells and the release of tumor antigens into the tumor space. Panitumumab, having an IgG2 backbone with very low affinity for CD16, is not capable of mediating ADCCs via NK cells. However, the clinical significance of this difference remains a matter of debate; the results of comparative studies did not show a clear advantage of any of the drugs [40]. A randomized ASPECCT (Phase III) trial compared panitumumab to cetuximab in patients with chemotherapy-resistant mCRC with wild-type *KRAS* exon 2. Treatment with one substance over another has not been demonstrated, but a clinically significant advantage of panitumumab is the lower risk of hypersensitivity reactions due to wholly human origin [41]. Although the PRIME study established that panitumumab is the first-line option for *RAS* WT tumors, subsequent evidence clearly showed that tumor location significantly modifies treatment benefit, fundamentally changing patient selection in routine clinical practice. Anti-EGFR monoclonal antibody therapy has shown a survival benefit primarily in patients with left-sided bowel tumors with wild-type *RAS*. Therefore, before incorporating this therapeutic strategy, it is essential to assess both the molecular status and the tumor location [42].

## 6. Panitumumab—Clinical Data

The clinical evidence supporting the FDA approval of panitumumab in 2006 included a randomized phase III trial comparing panitumumab plus best supportive care (BSC) with BSC alone in 463 patients with chemotherapy-refractory mCRC. The results of this trial were subsequently published by Van Cutsem et al. in 2007 [43]. Panitumumab significantly prolonged progression-free survival (HR 0.54; 95% CI 0.44–0.66; *p* < 0.0001). A retrospective analysis of this study showed that this benefit was primarily observed in patients with wild-type *KRAS*.

In the first-line treatment, the key evidence of effectiveness comes from the PRIME study. In the final analysis of this randomized phase III trial, adding panitumumab to the FOLFOX4 regimen in the wild-type *RAS* group increased median overall survival by 5.6 months (25.8 vs. 20.2 months) compared with FOLFOX4 alone. The PRIME study also confirmed that full genotyping of *RAS*, not only *KRAS* exon 2, is necessary for proper patient qualification, as mutations in other *KRAS* and *NRAS* exons were associated with a lack of benefit from therapy [44].

More recent evidence supporting the first-line use of panitumumab comes from the phase III PARADIGM trial, which directly compared panitumumab plus mFOLFOX6 with bevacizumab plus mFOLFOX6 in patients with chemotherapy-naïve, *RAS* wild-type, unresectable mCRC. The primary analysis was hierarchically focused on patients with left-sided primary tumors. In this population, panitumumab significantly improved median overall survival compared with bevacizumab (37.9 vs. 34.3 months; HR 0.82; 95.798% CI, 0.68–0.99; *p* = 0.03), although median progression-free survival was similar between the groups (13.1 vs. 11.9 months; HR 1.00; 95% CI, 0.83–1.20). Panitumumab was also associated with a higher objective response rate (80.2% vs. 68.6%) and a higher curative resection rate (18.3% vs. 11.6%) in patients with left-sided tumors [45]. Importantly, the benefit was not reproduced in patients with right-sided tumors, in whom median overall survival was 20.2 months with panitumumab and 23.2 months with bevacizumab. These findings provide prospective evidence that primary tumor sidedness is an important determinant of treatment selection and support panitumumab plus doublet chemotherapy as a preferred first-line strategy in appropriately selected patients with RAS wild-type, left-sided mCRC.

In the second line of treatment, the efficacy of the panitumumab + FOLFIRI regimen was documented. In the final analysis of the 20050181 study (Peeters et al.), it was shown that in the wild-type *KRAS* group, the addition of panitumumab to FOLFIRI significantly improved progression-free survival (median 6.7 vs. 4.9 months; HR 0.82; *p* = 0.023), and the response rate increased from 10% to 36% [46]. Beyond prolonging disease control, the high response rates achieved with panitumumab-based combinations may have particular clinical value in conversion therapy. This strategy aims to achieve sufficient tumor shrinkage to enable potentially curative local treatment in patients with initially unresectable metastatic disease, particularly those with liver-limited metastases. In the phase II PLANET-TTD trial (NCT00885885), first-line panitumumab combined with either FOLFOX4 or FOLFIRI produced high objective response rates in patients with multiple or unresectable liver-limited metastases. Surgical resection was subsequently performed in 45% and 59% of patients, respectively; among patients with RAS wild-type tumors, the corresponding resection rates were 37% and 69%. R0–R1 resection was achieved in 34% and 46% of the overall population and in 26% and 54% of patients with RAS wild-type disease, respectively [47]. Importantly, early tumor shrinkage and greater depth of response were associated with higher resectability rates, highlighting that the clinical value of panitumumab in this setting extends beyond conventional survival endpoints and may include facilitating potentially curative surgery in appropriately selected patients [47]. These findings support early multidisciplinary reassessment of initially unresectable liver-limited disease during highly active panitumumab-based treatment.

Earlier evidence from the MetaPan study also supported the conversion potential of panitumumab-based therapy. Among patients with *KRAS* wild-type disease treated with XELOX plus panitumumab, an objective response rate of 65% was achieved, and 15 patients with initially unresectable liver metastases became eligible for surgical resection [48].

In a direct comparison with cetuximab, the ASPECCT study, a randomized, open-label, phase III study in 1010 patients, demonstrated no superiority of panitumumab over cetuximab in terms of overall survival in patients with chemo-refractory mCRC with wild-type *KRAS* exon 2. Both drugs showed comparable efficacy, differing primarily in their tolerance profile. As previously mentioned, panitumumab was less likely to cause severe infusion reactions [41].

An important factor modifying the response to anti-EGFR therapy is the location of the primary tumor. Retrospective analyses of four randomized panitumumab trials showed that patients with *RAS* WT and left-sided tumors had a clear benefit from anti-EGFR treatment, whereas outcomes are less favorable in right-sided disease [49]. These observations have subsequently been strengthened by prospective evidence from the PARADIGM trial, which demonstrated an overall survival advantage for panitumumab over bevacizumab in patients with RAS wild-type, left-sided mCRC, but not in those with right-sided primary tumors [45]. The key clinical trials defining the efficacy and evolving clinical role of panitumumab in mCRC are summarized in Table 3.

### Safety and Management of Panitumumab-Related Adverse Events

Panitumumab has a well-characterized toxicity profile, with dermatologic adverse events—including acneiform rash, xerosis, pruritus, and paronychia—being the most common treatment-related toxicities. Proactive management is important to maintain treatment tolerability. In the randomized phase II STEPP trial, a pre-emptive regimen including moisturizers, sunscreen, topical corticosteroids, and doxycycline reduced the incidence of grade ≥2 skin toxicity from 62% to 29% compared with reactive treatment [51].

Hypomagnesemia is another clinically relevant toxicity of panitumumab; therefore, serum magnesium, calcium, and potassium should be monitored during treatment and supplemented when necessary [52]. In the ASPECCT trial, grade 3–4 infusion reactions were less frequent with panitumumab than with cetuximab (<0.5% vs. 2%), whereas grade 3–4 hypomagnesemia was more frequent (7% vs. 3%) [46]. Overall, early dermatologic prophylaxis, electrolyte monitoring, and appropriate treatment modification in patients with severe toxicity are important components of safe panitumumab therapy [51,52].

## 7. Therapeutic Strategies in Clinical Practice

Modern mCRC treatment is based on multi-stage molecular qualification. Assessment of MSI/dMMR, *RAS*, *BRAF*, and HER2 status should be completed prior to initiating any systemic treatment. The first step in treatment selection is to assess the MSI/dMMR status. Current guidelines from the NCCN, ESMO (European Society for Medical Oncology) and CSCO (Chinese Society of Clinical Oncology) prefer front-line checkpoint inhibitors in patients with the MSI-H/dMMR phenotype. In patients with MSS/pMMR, *BRAF* status is then assessed. In this group, breakthrough data were shown by the BREAKWATER study—the encorafenib + cetuximab + mFOLFOX6 regimen significantly improves objective response rates (60.9% vs. 40.0%; *p* = 0.0008) compared to standard chemotherapy, with a median response duration of 13.9 months [53]. The analysis also showed a significant improvement in median PFS (12.8 vs. 7.1 months; HR 0.53) and OS benefit (overall survival), which led to the FDA registering the regimen in the first-line treatment [54]. In patients without *BRAF* mutations, it is important to assess the status of *RAS*. In the case of *RAS* mutations, anti-EGFR therapy is contraindicated, and two-drug or triple-drug regimens with bevacizumab remain the standard. Bevacizumab is a recombinant humanized monoclonal antibody directed against vascular endothelial growth factor (VEGF-A). By neutralizing VEGF, it inhibits angiogenesis, thus limiting its growth and ability to metastasize [55]. Unlike panitumumab or cetuximab, its effectiveness does not depend on the status of the *RAS* or *BRAF* mutation, so it remains a therapeutic option also in patients with *RAS* mutations for whom anti-EGFR therapy is contraindicated [56]. In patients with wild-type *RAS* and *BRAF* and left-sided tumor localization, anti-EGFR therapy, including panitumumab in combination with chemotherapy, is recommended [57].

According to NCCN guidelines, panitumumab in combination with FOLFOX or FOLFIRI is recommended as a first-line option in patients with *RAS*/*BRAF* WT mCRC. As discussed earlier, PRIME confirmed an increase in the median OS by 5.6 months compared to FOLFOX4 alone. In the second-line setting, for patients previously treated with oxaliplatin, panitumumab or cetuximab in combination with FOLFIRI is an option recommended by ESMO in selected patients with left-sided, *RAS*/*BRAF* WT tumor who have not previously received anti-EGFR therapy [57]. In some patients who have benefited from anti-EGFR in previous lines and subsequently underwent alternative treatment regimens, it is possible to re-initiate panitumumab—a strategy referred to as rechallenge. The biological rationale for the anti-EGFR reactivation strategy is the observation that resistant clones selected during treatment may disappear after treatment discontinuation, restoring sensitivity to EGFR blockade. A detailed discussion of this strategy and the CHRONOS data is provided in Section 8.2 [34]. A simplified biomarker-guided framework for therapeutic decision-making and the clinical positioning of panitumumab in mCRC is presented in Figure 3.

## 8. Anti-EGFR Therapy Resistance and Strategies for Overcoming It

Resistance to anti-EGFR therapy is divided into primary resistance, present before the start of treatment, and acquired resistance, developing during it. The mechanisms of primary resistance include *BRAF* V600E mutations and changes in the *MET*, *PIK3CA*, *PTEN* and HER2 genes. In contrast, acquired resistance is mainly determined by *EGFR* ectodomain mutations, *MET* amplification, *BRAF* V600E mutations and HER2 amplification [35]. Studies indicate that the acquired resistance in a significant proportion of cases (40–50%) results from the appearance of changes in the MAPK pathway: *KRAS*, *NRAS*, *BRAF*, *MAP2K1* and *EGFR*-ECD mutations, as well as *ERBB2* and *MET* amplification. HER2 amplification can be both a primary and an acquired mechanism. This is likely due to the expansion of pre-existing rare HER2-amplified clones under the selection pressure of EGFR inhibitors [35].

### 8.1. Monitoring Resistance by ctDNA

Analysis of circulating tumor DNA (ctDNA) enables non-invasive, real-time tracking of tumor clonal evolution, which is crucial in the context of anti-EGFR resistance. *RAS* mutations and HER2 and *MET* amplifications are the most commonly detected mechanisms of resistance in both tissue and plasma. Importantly, resistance-associated alterations may decline after discontinuation of anti-EGFR therapy, potentially identifying patients who regain sensitivity to EGFR blockade and may therefore become candidates for rechallenge [36]. Dynamic ctDNA analysis enables identification of molecular mechanisms of progression and longitudinal monitoring of resistant clones. The decline or disappearance of resistance-associated alterations after discontinuation of anti-EGFR therapy may identify patients who could regain sensitivity to EGFR blockade and therefore become candidates for rechallenge [36].

### 8.2. Post-Progression Strategies—Panitumumab Rechallenge

As discussed in the previous section, the biological justification for the reintroduction of anti-EGFR is the disappearance of resistant clones after discontinuation of treatment. The CHRONOS study provides proof-of-concept evidence for the feasibility of rational, fluid biopsy-based patient selection for this strategy. Patients with tissue-wild-type *RAS* who had prior anti-EGFR therapy underwent interventional ctDNA screening. Of the 52 patients evaluated, 16 (31%) carried resistance mutations and were excluded. In the group of 27 enrolled, 30% objective responses and 63% disease control were achieved, which compares favorably with standard tertiary therapies. These results confirm that ctDNA may act as a qualifying tool for rechallenge, limiting exposure to ineffective treatment in patients with persistent resistance mutations [34]. However, CHRONOS was a small, single-arm phase II study, and ctDNA-guided panitumumab rechallenge has not yet become a universal standard of care.

## 9. Perspectives and Directions of Research

### 9.1. Emerging Molecularly Targeted Combinations Involving EGFR Blockade

One of the most actively studied directions is the association of *BRAF* and MEK inhibitors with EGFR blockade. Initial evaluations of the dabrafenib + trametinib + panitumumab regimen showed a higher response rate in the triple combination arms (21%) than dabrafenib plus panitumumab alone (10%) or trametinib plus panitumumab (0%) [58].

The therapeutic relevance of *EGFR* blockade in *BRAF* V600E-mutated mCRC has been further defined by trials combining inhibition of *BRAF* with *EGFR*. The phase III BEACON CRC trial established encorafenib plus cetuximab as an effective targeted strategy in previously treated *BRAF* V600E-mutated mCRC, demonstrating that simultaneous inhibition of *BRAF* and *EGFR* can overcome, at least in part, adaptive MAPK pathway reactivation [59]. More recently, the phase III BREAKWATER trial extended this strategy to the first-line setting. In patients with previously untreated *BRAF* V600E-mutated mCRC, encorafenib plus cetuximab and mFOLFOX6 significantly improved objective response rate (60.9% vs. 40.0%; odds ratio, 2.443; *p* = 0.0008) and progression-free survival (median, 12.8 vs. 7.1 months; HR 0.53, 95% CI 0.41–0.68; *p* < 0.001) compared with standard therapy [54]. Although both BEACON and BREAKWATER evaluated cetuximab rather than panitumumab, these findings provide important proof of principle that EGFR blockade can retain therapeutic relevance in *BRAF* V600E-mutated disease when combined with inhibition of the oncogenic driver. However, these results should not be directly extrapolated to panitumumab-containing regimens, which require separate clinical validation.

An important recent development concerns patients with *KRAS G12C*-mutated mCRC, in whom conventional anti-EGFR therapy is ineffective because of constitutive downstream *RAS* signaling. However, inhibition of *KRAS G12C* may induce adaptive *EGFR*-mediated pathway reactivation, providing a biological rationale for simultaneous *KRAS G12C* and *EGFR* blockade. In the phase Ib CodeBreaK 101 study, sotorasib combined with panitumumab demonstrated clinically meaningful activity in chemotherapy-refractory *KRAS G12C*-mutated mCRC, with a confirmed objective response rate of 30.0%, median progression-free survival of 5.7 months, and median overall survival of 15.2 months [60]. These findings were subsequently confirmed in the randomized phase III CodeBreaK 300 trial, in which sotorasib 960 mg once daily plus panitumumab significantly improved median progression-free survival compared with standard treatment (5.6 vs. 2.0 months; HR 0.48, 95% CI 0.30–0.78), with an objective response rate of 26.4% versus 0%, respectively [61]. These results represent an important evolution of the conventional anti-EGFR treatment paradigm: although *RAS* mutations remain predictive of resistance to panitumumab used as conventional *EGFR*-directed therapy, *KRAS G12C* represents a molecularly defined subgroup in which panitumumab can regain therapeutic relevance when combined with direct inhibition of the oncogenic driver.

In patients with HER2 overexpression and wild-type *RAS*/*BRAF*, combining anti-HER2 therapy with anti-EGFR therapy remains a promising direction, although clinical data remain limited. In the case of severe infusion reactions to cetuximab observed in 2–5% of patients treated with this drug, switching to panitumumab in combination with encorafenib is a safe and effective alternative, as confirmed by an international multicenter AGEO group analysis of patients with *BRAF* V600E-mutated mCRC, who could not continue treatment with cetuximab [62].

### 9.2. Immunotherapy vs. Anti-EGFR Therapy, Synergy Potential

The effective integration of immunotherapy into the treatment of patients with MSS/pMMR mCRC remains a major clinical challenge. Available evidence suggests that anti-EGFR monoclonal antibodies, including cetuximab and panitumumab, may modulate antitumor immune responses, providing a biological rationale for combining EGFR blockade with immune checkpoint inhibitors. The phase II CAVE study evaluating cetuximab plus avelumab as a rechallenge strategy, predominantly in patients with MSS disease, reported a median OS of 18.6 months at long-term follow-up [63,64].

More directly relevant to panitumumab, in patients with refractory *KRAS*/*NRAS*/*BRAF* wild-type MSS mCRC, the phase II multicenter trial NCT03442569 evaluating panitumumab combined with nivolumab and ipilimumab demonstrated an ORR of 32% (95% CI, 21–45%) among 49 patients evaluable for the primary endpoint, with a median PFS of 6.0 months and a median OS of 17.4 months [65,66].

Although these findings suggest clinically meaningful activity in a population with limited sensitivity to immune checkpoint blockade alone, they derive from a single-arm phase II study and require confirmation in randomized trials. Therefore, combined EGFR and immune-checkpoint blockade should currently be regarded as an investigational strategy rather than an established treatment approach.

### 9.3. CCR5/Maraviroc and Other New Targets

As discussed in Section 4, the *CCL5*/*CCR5* axis creates an immunosuppressive tumor microenvironment and represents a potential therapeutic target. Ongoing research on maraviroc in combination with pembrolizumab (the PICASSO phase I study) opens the prospect of microenvironmental immune modulation as a complementary strategy to targeted therapies [33]. Among the further directions of development, in addition to the previously discussed *CCR5* axis, the *KRAS* G12C mutation and liquid ctDNA biopsy, the areas of artificial intelligence and adaptive therapy are attracting increasing attention. AI models are being increasingly studied in mCRC as tools to support the prediction of responses to targeted therapies. It is also beginning to be used to assess metastasis risk and classify MSI directly from histopathological images. Models based on radiomics, i.e., the analysis of tumor imaging features on computed tomography, show moderate predictive power for chemotherapy response in patients with liver metastases. Despite promising results, most of these tools still require large-scale clinical validation before being implemented into routine practice [67].

Adaptive therapy, on the other hand, is a treatment concept based on evolutionary principles: it assumes that maintaining a pool of cancer cells that remain sensitive to treatment allows limiting the expansion of resistant clones through intercellular competition, rather than striving for a one-time, maximum elimination of the tumor. In practice, this means intermittent or dynamically adjusted drug dosing based on the current tumor size, rather than continuous treatment at the maximum tolerated dose [68]. This concept is already being clinically tested in the context of panitumumab itself. The IMPROVE study compares intermittent and continuous administration of panitumumab in combination with FOLFIRI as first-line treatment for patients with wild-type *RAS*/*BRAF*, based on the same biological observation that justifies the rechallenge strategy described earlier [69].

### 9.4. Precision Medicine and Liquid Biopsy

Beyond its role in identifying resistance mechanisms and selecting candidates for anti-EGFR rechallenge, ctDNA may facilitate longitudinal assessment of tumor evolution in mCRC. However, its routine integration into panitumumab treatment decisions requires further methodological standardization and prospective clinical validation [16,19].

#### Limitations and Future Directions of ctDNA-Guided Monitoring

Despite its potential, ctDNA should currently complement rather than replace tissue-based molecular testing. Liquid biopsy is minimally invasive, can be repeated longitudinally, and may better capture spatial and temporal tumor heterogeneity; however, low tumor shedding and certain metastatic patterns may result in false-negative findings [17,70]. Broader clinical implementation is further limited by inter-assay variability, incomplete standardization of analytical procedures and detection thresholds, cost, and unequal access to validated platforms [70,71]. Tissue biopsy therefore remains important, particularly when plasma results are inconclusive. Although CHRONOS provides proof-of-concept for ctDNA-guided panitumumab rechallenge, prospective validation of standardized assays and clinically relevant molecular thresholds is required before this strategy can be routinely implemented [70].

### 9.5. New Strategies

Leronlimab is also a promising therapeutic candidate against CCR5. It is a humanized IgG4 monoclonal antibody that blocks the CCR5 receptor. In a humanized mouse model of colon cancer xenograft, leronlimab showed significant anti-cancer activity—a reduction in lung metastases by approximately 87% and a reduction in tumor vascularity by 62%. Importantly, the anti-cancer effect disappeared in models without humanized leukocytes, indicating a key role of immune cells in the drug’s mechanism of action. CCR5 and its ligands have further been observed to be overexpressed in human colon cancer, correlating with increased expression of immune checkpoint genes in both MSS and MSI tumors [11]. Leronlimab is currently being evaluated in the phase II CLOVER trial (NCT06699836) in combination with trifluridine/tipiracil (TAS-102) and bevacizumab in patients with previously treated MSS mCRC. Preliminary results reported in 2026 showed that the combination was feasible and well tolerated, with early clinical and biomarker activity; however, these findings remain preliminary and require confirmation with mature efficacy data [72].

Taken together, these findings support further investigation of CCR5-directed modulation of the tumor immune microenvironment as a potential therapeutic strategy in treatment-refractory MSS mCRC. However, the preclinical evidence cited above derives from a preprint that has not yet undergone peer review (Lindner et al., 2025) [11] and should therefore be regarded as preliminary until independently validated in peer-reviewed studies and clinical trials.

## 10. Conclusions

Metastatic colorectal cancer remains a serious therapeutic challenge. Still, the rapid development of targeted therapies and the deepening understanding of the molecular biology of cancer significantly improve patient prognosis. Panitumumab is one of the few medicines for which robust data have been collected from phase III studies covering subsequent lines of treatment (Van Cutsem study, PRIME, 20050181). Its clinical role is well established in appropriately selected patients with *RAS* wild-type, particularly left-sided mCRC, while contemporary molecular stratification increasingly defines the settings in which EGFR blockade is most likely to provide clinical benefit. Comprehensive molecular profiling, including *RAS* and *BRAF* testing, MSI/dMMR status, HER2 amplification, and primary tumor sidedness, has substantially improved patient selection for targeted treatment. Nevertheless, current biomarker-guided strategies remain imperfect: a proportion of molecularly eligible patients do not respond to panitumumab, while intratumoral heterogeneity and clonal evolution under therapeutic pressure may limit the predictive value of a single baseline molecular assessment. ctDNA provides a potential means of dynamically monitoring tumor evolution and acquired resistance and may support selection for anti-EGFR rechallenge. However, evidence supporting ctDNA-guided panitumumab rechallenge remains limited, and the CHRONOS study should be regarded as proof-of-concept rather than definitive evidence for routine implementation [34].

Among the emerging therapeutic strategies, three research directions appear particularly relevant for further defining the role of panitumumab in mCRC. First, molecularly selected combination strategies should be prioritized, particularly simultaneous blockade of EGFR and actionable downstream drivers. The clinical activity of sotorasib plus panitumumab in *KRAS* G12C-mutated mCRC provides strong evidence that panitumumab may retain therapeutic relevance even in selected *RAS*-mutant tumors when the oncogenic driver is directly inhibited [60,61]. Further studies should determine which additional molecular subgroups may benefit from analogous vertical pathway inhibition strategies.

Second, ctDNA-guided anti-EGFR rechallenge represents a promising but not yet fully mature strategy for routine clinical implementation. The CHRONOS study demonstrated that ctDNA can identify patients without detectable resistance alterations who may regain sensitivity to panitumumab [34]. However, prospective randomized studies using standardized assays, predefined molecular thresholds, and clinically validated sampling intervals are required before ctDNA-guided rechallenge can be considered a universal standard of care.

Third, combining panitumumab with immune checkpoint inhibition warrants further investigation, particularly in MSS/pMMR mCRC, where effective immunotherapy remains an unmet clinical need. The observed activity of panitumumab combined with nivolumab and ipilimumab provides a rationale for further evaluation [66], but the available evidence remains limited and should be confirmed in randomized trials with biomarker-driven patient selection.

Taken together, future research should move beyond evaluating panitumumab as an isolated EGFR inhibitor and instead define its role within dynamically selected, molecularly rational combination strategies. Integration of comprehensive genomic profiling with longitudinal molecular assessment may ultimately refine treatment selection; however, biomarker-guided strategies require further prospective validation, methodological standardization, and demonstration of clinical utility before they can be universally incorporated into panitumumab management.

## Figures and Tables

**Table 1:** Current recommendations for molecular biomarker testing in patients with metastatic colorectal cancer (mCRC) according to NCCN (Version 5.2025), ESMO (2023), and CSCO (2024 update) guidelines [8,9,10].

Biomarker	NCCN	ESMO	CSCO
*RAS* (*KRAS*/*NRAS*)	Test in all patients; required for anti-EGFR selection	Test *KRAS*/*NRAS* exons 2–4; mandatory before anti-EGFR therapy	Recommended for molecular work-up and anti-EGFR selection
*BRAF* V600E	Test in all patients; prognostic and predictive relevance	Test with *RAS* at diagnosis	Recommended; guides *BRAF*-targeted therapy
MMR/MSI	Test in metastatic disease; guides immunotherapy	Test at mCRC diagnosis; guides immunotherapy	Recommended; guides immunotherapy
HER2	Test as an actionable biomarker in appropriate patients	Recommended in *RAS* WT disease	Consider in selected patients for anti-HER2 therapy
ctDNA/liquid biopsy	May complement tissue-based molecular testing	May be used when adequate tissue is unavailable	Not established for routine standalone testing

**Table 2:** Clinical relevance of molecular biomarkers in metastatic colorectal cancer [7,16,17,26,29,30,31,34,35,36].

Category	Biomarkers	Clinical Application
Diagnostic/molecular profiling	*RAS* (*KRAS*/*NRAS*), *BRAF*, MSI/dMMR, HER2	Confirmation of the tumor’s molecular profile before treatment qualification
Predictive	*RAS*, *BRAF* V600E, MSI-H/dMMR, HER2, *NTRK* fusions	Guides treatment selection
Prognostic	BRAF V600E, MSI/dMMR, primary tumor sidedness	Indicates disease course and expected prognosis independent of treatment
Dynamic monitoring	ctDNA, including emerging *RAS*, *BRAF*, *EGFR*, HER2 and *MET* resistance alterations	Tracking clonal evolution over time, detecting acquired resistance, qualification for rechallenge (CHRONOS)
Experimental/emerging targets	CCR5/CCL5 axis (selected tumor microenvironment biomarkers)	Modulation of the tumor immune microenvironment; early, preclinical data

**Table 3:** Key clinical trials evaluating panitumumab-based treatment strategies in metastatic colorectal cancer [34,41,44,45,46,47,50].

Study	Phase	Molecular Population	Treatment	Key Efficacy Results	Main Clinical Relevance
PRIME [44]	Phase III; first-line	*RAS* WT mCRC	Panitumumab + FOLFOX4 vs. FOLFOX4	PFS: 10.1 vs. 7.9 months; HR 0.72. OS: 25.8 vs. 20.2 months; HR 0.78	Established first-line efficacy in RAS WT mCRC
PARADIGM [45]	Phase III; first-line	*RAS* WT, left-sided mCRC	Panitumumab + mFOLFOX6 vs. bevacizumab + mFOLFOX6	OS: 37.9 vs. 34.3 months; HR 0.82. ORR: 80.2% vs. 68.6%	Supports first-line panitumumab in left-sided RAS WT mCRC.
20050181 [46]	Phase III; second-line	*KRAS* exon 2 WT mCRC	Panitumumab + FOLFIRI vs. FOLFIRI	PFS: 6.7 vs. 4.9 months; ORR: 36% vs. 10%. OS: 14.5 vs. 12.5 months (NS)	Improved PFS and ORR in second-line treatment.
ASPECCT [41]	Phase III; chemotherapy-refractory	*KRAS* exon 2 WT mCRC	Panitumumab vs. cetuximab	Final OS: 10.2 vs. 9.9 months; HR 0.94	Non-inferior to cetuximab for OS.
PLANET-TTD [47]	Phase II; first-line, liver-limited disease	*KRAS* WT; exploratory *RAS* WT analysis	Panitumumab + FOLFOX4 vs. panitumumab + FOLFIRI	ORR: 74% vs. 67%; resection: 45% vs. 59%	Supports panitumumab-based conversion therapy.
CHRONOS [34]	Phase II; anti-EGFR rechallenge	Tissue *RAS* WT; ctDNA negative for *RAS*/*BRAF*/*EGFR* resistance mutations	Panitumumab rechallenge	ORR: 30%; DCR: 63%	Proof-of-concept for ctDNA-guided rechallenge.
TRIPLETE [50]	Phase III; first-line	*RAS*/*BRAF* WT mCRC	mFOLFOXIRI + panitumumab vs. mFOLFOX6 + panitumumab	OS: 41.1 vs. 33.3 months; HR 0.79; no significant PFS or ORR improvement	OS benefit without significant PFS or ORR improvement.

## Data Availability

The original contributions presented in this study are included in the article. Further inquiries can be directed to the corresponding author.

## Abbreviations

The following abbreviations are used in this manuscript:

*ADCC*	antibody-dependent cellular cytotoxicity
*APC*	adenomatous polyposis coli
*BRAF*	B-Raf proto-oncogene serine/threonine-protein kinase
*BSC*	best supportive care
*CI*	confidence interval
*CIMP*	CpG island methylator phenotype
*CIN*	chromosomal instability
*CRC*	colorectal cancer
*CCR5*	C-C chemokine receptor type 5
*CCL5*	C-C motif chemokine ligand 5
*ctDNA*	circulating tumor DNA
*CSCO*	Chinese Society of Clinical Oncology
*CTLA-4*	cytotoxic T-lymphocyte-associated protein 4
*dMMR*	deficient mismatch repair
*EGFR*	epidermal growth factor receptor
*ERBB2*	erb-b2 receptor tyrosine kinase 2
*ESMO*	European Society for Medical Oncology
*FDA*	Food and Drug Administration
*FOLFIRI*	scheme: fluorouracil, leucovorin, irinotecan
*FOLFOX*	scheme: fluorouracil, leucovorin, oxaliplatin
*HER2*	human epidermal growth factor receptor 2
*HGF*	hepatocyte growth factor
*HR*	hazard ratio
*IgG*	immunoglobulin G
*JAK/STAT*	Janus kinase/signal transducer and activator of transcription
*KRAS*	Kirsten rat sarcoma viral proto-oncogene
*MAPK*	mitogen-activated protein kinase
*mCRC*	metastatic colorectal cancer
*MDSC*	myeloid-derived suppressor cells
*MEK*	mitogen-activated protein kinase
*MET*	mesenchymal–epithelial transition factor
*MMR*	mismatch repair
*MSI*	microsatellite instability
*MSI-H*	microsatellite instability-high
*MSI-L*	microsatellite instability-low
*MSS*	microsatellite stable
*NCCN*	National Comprehensive Cancer Network
*NRAS*	neuroblastoma RAS viral proto-oncogene
*NTRK*	neurotrophic tyrosine receptor kinase
*ORR*	objective response rate
*OS*	overall survival
*PD-1*	programmed cell death protein 1
*PD-L1*	programmed death-ligand 1
*PFS*	progression-free survival
*PI3K*	phosphatidylinositol 3-kinase
*pMMR*	proficient mismatch repair
*PTEN*	phosphatase and tensin homolog
*RAS*	rat sarcoma virus
*TMB*	tumor mutational burden
*TP53*	tumor protein p53
*TRK*	tropomyosin receptor kinase
*WT*	wild type
